# Neutrophils in Myocarditis: A Focus on the Secretory and Phagocytotic Functions

**DOI:** 10.31083/RCM39207

**Published:** 2025-08-27

**Authors:** Lisha Jia, Yuqing Shen, Wei Feng, Rui Mai, Xianwei Wang

**Affiliations:** ^1^Human Anatomy Laboratory, School of Basic Medicine, Xinxiang Medical University, 453003 Xinxiang, Henan, China; ^2^Henan Key Laboratory of Medical Tissue Regeneration, Xinxiang Medical University, 453003 Xinxiang, Henan, China; ^3^Coronary Care Unit, The Third Clinical College of Xinxiang Medical University, Xinxiang Medical University, 453003 Xinxiang, Henan, China; ^4^Department of Human Anatomy and Histoembryology, Xinxiang Medical University, 453003 Xinxiang, Henan, China

**Keywords:** inflammation, neutrophil extracellular traps (NETs), innate immune response, therapeutics, chemokine, cytokine

## Abstract

Myocarditis is a life-threatening inflammatory disorder that affects the cardiac muscle tissue. Current treatments merely regulate heart function but fail to tackle the root cause of inflammation. In myocarditis, the initial wave of inflammation is characterized by the presence of neutrophils. Subsequently, neutrophils secrete chemokines and cytokines at the site of heart tissue damage to recruit additional immune cells and regulate defense responses, thereby exacerbating myocarditis. Recent discoveries showing neutrophil extracellular traps (NETs) and their components not only reinforce the proinflammatory functions of neutrophils, inducing enhanced interleukin (IL)-8 secretion, but also induce monocyte/macrophage activation, differentiation, and phagocytic function through the inflammasome pathway. The inflammasome cascade triggers a positive feedback loop through the secretion of proinflammatory cytokines, which leads to further neutrophil activation and degranulation, NET release, monocyte and macrophage infiltration, tissue degradation, and myocardial damage, indicating that neutrophils promote myocarditis-induced cardiac necrosis and an anti-cardiac immune response. In addition, neutrophils can induce oxidative stress and damage cellular structures by releasing excess reactive oxygen species (ROS), thus exacerbating tissue damage in myocarditis. Meanwhile, the recruitment of cells, which is facilitated by neutrophil-secreted chemokines, and the consumption of cells through neutrophil phagocytosis can form a closed loop that continuously maintains a proinflammatory state. This review summarizes the role of neutrophil secretion, phagocytosis and their relationship in myocarditis, and discusses the function of certain agents, such as chemokine antagonists, midkine blockers and neutrophil peptidyl arginine deiminase 4 (PAD4) inhibitors in inhibiting neutrophil secretion and phagocytosis, to provide perspective for myocarditis treatments through the inhibition of neutrophil secretion and phagocytosis.

## 1. Introduction

Myocarditis, caused by viruses, bacteria, fungi, parasites, radiation, and toxic pollutants, is an inflammatory disease of the myocardium characterized by the infiltration of immune cells into the myocardium. Myocarditis is serious and harmful, often occurs in young and middle-aged adults, and has a high mortality rate [1]. The global annual incidence of myocarditis is approximately 1.5–4.2 per 100,000, while the actual incidence may be as high as 22 per 100,000 (with a high rate of missed diagnoses) [2]. Among them, patients aged 20–40 years account for more than 50%, and the risk of male patients is 1.7 times higher than that of female patients. The mortality of fulminant myocarditis is as high as 50–70% [3]. In 20–30% of cases, myocarditis will progress to dilated cardiomyopathy, with the 10-year mortality rate rising to 30–50% [4]. Despite significant advances in myocarditis-related research, the pathogenesis of myocarditis remains unclear. However, inflammation is a significant driver of both the occurrence and development of myocarditis. Moreover, the activation of various immune cells is closely related to the occurrence of myocarditis [5]. Notably, neutrophils have a unique role in the development and progression of myocarditis, but large gaps in knowledge remain. Further, existing studies have yet to elucidate the spatiotemporal distribution patterns of myocarditis-specific neutrophil subsets; meanwhile, only the function of the N1 proinflammatory subtype has been preliminarily confirmed, and the pathological contributions of other subtypes remain unclear. Although the dependency of neutrophil extracellular trap (NET) formation on peptidyl arginine deiminase 4 (PAD4) has been confirmed, the fine regulatory network of NET formation in the myocardial microenvironment remains unclear. Existing neutrophil-related drugs have limitations, including low cardiac targeting efficiency and incomplete inhibition of neutrophils. Hence, improving our understanding of the role of neutrophils in the pathogenesis of myocarditis is of great significance for overcoming these difficult problems.

Neutrophils play an important role in myocarditis [6]. After passing through the vascular endothelium, neutrophils enter the myocardium, migrate further to the inflammatory focus, and eventually accumulate in the core area of pathogen infection or tissue injury. In the early stage of inflammatory cell infiltration, neutrophils secrete cytokines and phagocytose pathogens at the site of heart damage, and decrease cardiac monocyte recruitment and proinflammatory macrophage differentiation, improving myocarditis-induced cardiac necrosis [7]. Neutrophils are activated by pathogen-associated molecular patterns (PAMPs) and damage-associated molecular patterns (DAMPs) through pattern recognition receptors (PRRs), as well as DAMP receptors, and mediate phagocytosis and opsonization [8]. Neutrophil activity is initially beneficial; however, excessive activation of neutrophils may lead to detrimental effects [9]. When inflammation is recurrent or the inciting agent persists, neutrophils can synthesize and release chemokines and cytokines with proinflammatory activity, causing the recruitment of additional neutrophils, along with other immunocompetent cells, to the site of infection, thereby boosting the inflammatory response in myocarditis [10]. Neutrophils can also secrete and produce NETs that can capture, localize, and destroy pathogens [11].

**Fig. 1.**
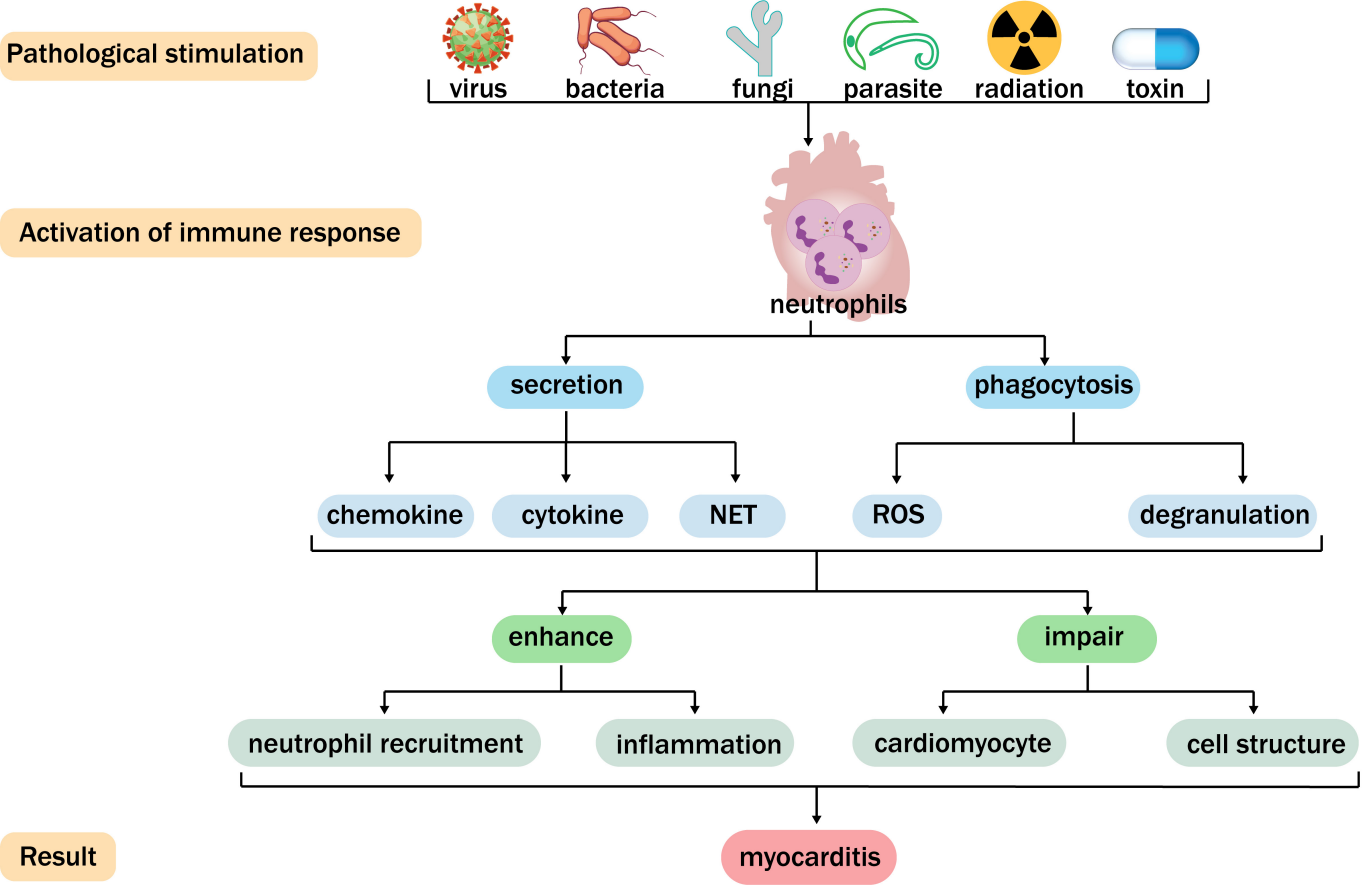
**Function of neutrophils in myocarditis**. Viruses, bacteria, fungi, parasites, radiation, and toxins can cause myocarditis and activate neutrophils. Neutrophils generate chemokines, cytokines, neutrophil extracellular traps (NETs), denudation, and reactive oxygen species (ROS) through secretion and phagocytosis. In this way, neutrophils can further enhance neutrophil recruitment and inflammation, damage cardiomyocytes and cellular structures, and subsequently facilitate the development of myocarditis.

When myocarditis occurs, NETs activate mononuclear/macrophage differentiation and phagocytosis through the inflammasome pathway and transfer autoantigens, including DNA and histones, to the host immune system, intensifying the inflammatory response and tissue damage. Additionally, upon recognition of invading pathogens, neutrophils will undergo phagocytosis and form phagosomes [12, 13]. Neutrophils release myeloperoxidase (MPO), neutrophil elastase (NE), nicotinamide adenine dinucleotide phosphate (NADPH), and other particles through intracellular degranulation to counter infection and generate reactive oxygen species (ROS) [14, 15]. However, uncontrolled degranulation results in excessive inflammation and oxidative stress, thereby exacerbating tissue damage in myocarditis (Fig. 1). Hence, neutrophils can be considered indispensable contributors to the development of myocarditis. This review aims to discuss the existing evidence for the role of neutrophils in the pathogenesis of myocarditis and describe recent insights, with a focus on phagocytosis and secretion. Next, we evaluate the potential of neutrophils as therapeutic targets in myocarditis.

## 2. Neutrophil Secretion

**Fig. 2.**
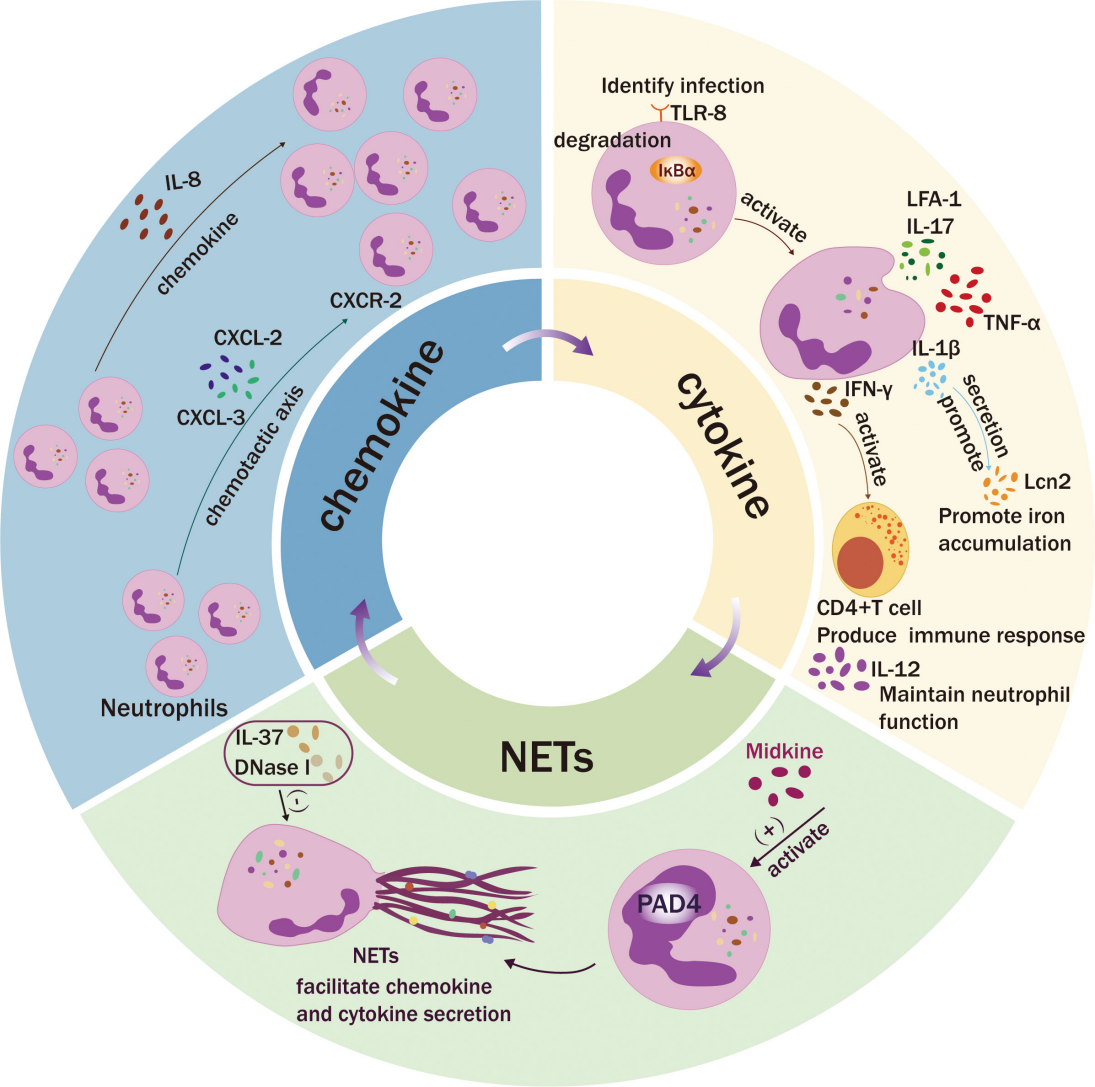
**Neutrophil secretion**. Neutrophils facilitate immune cell recruitment by secreting a diverse array of chemotactic factors and establishing a chemotactic gradient. After activation of Toll-like receptor (TLR)-8, cytokines such as interleukin (IL)-12, IL-1β, interferon-γ (IFN-γ), and other chemokines are secreted, triggering the immune response and initiating myocarditis. Subsequently, midkine (MK) and ADAMTS-1 activate neutrophils, and nuclear peptidyl arginine deiminase 4 (PAD4) promotes the formation of NETs, enhances the proinflammatory function of neutrophils, and accelerates the progression of myocarditis. IL-37 and DNase 1 can reduce the burden on the heart by inhibiting the formation of NETs. CXCR, C-X-C chemokine receptor; CXCL, C-X-C motif chemokine ligand; LFA-1, lymphocyte function-associated antigen-1; TNF-α, tumor necrosis factor-alpha.

Neutrophils act as the first responders at sites of injury to coordinate the initial proinflammatory response, and are capable of secreting a diverse array of substances, including chemokines, cytokines, and NETs [16]. These secreted substances facilitate the recruitment of additional neutrophils, modulate immune responses, and promote the prompt resolution of inflammation. The release of these secreted substances is stringently regulated to ensure that the substances function precisely at the appropriate time and location [17]. Secretion by neutrophils enables these substances to gather rapidly at the site of inflammation, activate various immune cells, and assume an exceptionally crucial role in combating infection and facilitating tissue repair. Nevertheless, unregulated cytokine secretion by neutrophils can result in excessive neutrophil aggregation, the release of proinflammatory cytokines, increased inflammation, and imbalanced immune regulation, ultimately promoting severe myocarditis (Fig. 2) [18].

### 2.1 Secretion of Chemokines Promotes Neutrophil Recruitment in Myocarditis 

Neutrophils accumulate in the heart at the site of tissue damage, where inflammatory signals are abundant, along a gradient of chemokines, such as interleukin (IL)-8, which is consistent with the well-known nature of rapid chemotaxis and migration to acutely injured tissue [19]. Clinical studies have shown that the levels of chemokines in patients with myocarditis are significantly elevated, with levels being closely related to the severity of the disease, inflammatory cell infiltration, and impaired cardiac function. Expression of the C-X-C motif chemokine ligand (CXCL)8 increases in patients with myocarditis and can efficiently attract neutrophils to migrate to the site of myocardial injury [14, 20]. The chemokine (C-C motif) ligand 2 (CCL2) expression level in the myocardium of patients with dilated cardiomyopathy (DCM) is higher than that in normal myocardial tissue, correlating with the degree of impaired cardiac function [21]. Furthermore, serum CCL2 levels in patients with acute myocarditis in the early stage of the disease are already higher than in healthy individuals. Moreover, the serum CCL2 level was even higher in patients who died from acute myocarditis. Therefore, high expression of CCL2 may aggravate myocardial injury by promoting the infiltration and activation of neutrophils, thereby affecting the prognosis of patients [22]. In myocarditis, the upregulation of chemokine genes is mainly driven by specific kinases and transcription factors, which aggravate myocardial injury by regulating immune cell infiltration and inflammatory cascades. In immune-related myocarditis, CXCL9+ fibroblast subsets highly express chemokines such as CXCL9 and CXCL10, and these cells are enriched in the Janus kinase-signal transducers and activators of transcription (JAK–STAT) signaling pathway. Indeed, activation of the JAK–STAT pathway can induce the transcription of chemokine genes, thereby enhancing the recruitment of immune cells [23]. Nuclear factor kappa-light-chain-enhancer of activated B (NF-κB) is a key inflammation-related transcription factor. In myocarditis, the activation of NF-κB is closely related to the upregulation of chemokines, including CCL2, CCL3, and CXCL2 [21]. Moreover, the levels of IL-8 secreted by neutrophils in patients with myocarditis are higher than in those without myocarditis [14].

Neutrophils clustered at sites of heart tissue damage acquire a profound proinflammatory phenotype, expressing high levels of chemokines that ceaselessly attract peripheral neutrophils to the heart [16], resulting in a positive feedback loop that promotes continuous neutrophil accumulation. Moreover, neutrophils are recruited to the site of heart tissue damage and develop a CXCL2/3-C-X-C chemokine receptor (CXCR) 2 self-chemotactic axis that causes peripheral neutrophils to migrate to the heart [10]. In a murine Coxsackievirus B3 (CVB3)-mediated myocarditis model, during infection, CVB3 upregulates the expression of chemokines (CXCL1, CXCL2, and CXCL3) in the heart, continuously attracting neutrophils from the peripheral blood [24]. Moreover, cell death from myocarditis leads to the release of DAMPs S100A8 and S100A9 [25], which are responsible for leukocyte migration (Fig. 2) [26]. S100A8 and S100A9 bind DAMP-sensing receptors, such as Toll-like receptors (TLRs), to cause increased cardiac inflammatory cell infiltration [27]. The association between S100A8 and S100A9 and DAMPs triggers a cascade of inflammatory responses, including the capturing of free-flowing neutrophils, by regulating rolling, adhesion, adhesion strengthening, intraluminal crawling, and transmigration, which leads to myocardial injury and exacerbates inflammation in myocarditis patients [28]. Neutrophils can also trigger an “inflammatory storm” by attracting and activating proinflammatory monocytes in the early stage of disease, therefore accelerating the early inflammatory response and later myocardial remodeling [29]. Post-translational modification (PTM) of chemokines also plays a key role in the recruitment of neutrophils. Proteolysis generates bioactive chemokine fragments with enhanced chemoattractant activity, while citrullination potentiates their capacity to mobilize neutrophils. In contrast, nitration negatively modulates the efficiency of chemokine signaling. Furthermore, glycosylation modifies chemokine–glycosaminoglycan binding interactions, thereby influencing the retention of these glycoproteins at inflammatory sites [30, 31]. These PTMs precisely regulate the functions of chemokines through multiple mechanisms, thereby influencing the recruitment of neutrophils and the progression of inflammatory responses. The early inhibition of self-recruited neutrophils could act as a crucial therapeutic target for myocarditis. Meanwhile, mice with CVB3-induced fulminant myocarditis (FM), when treated with CXCR2-neutralizing antibodies, exhibit reduced mortality and cardiac deterioration, indicating that blocking CXCR2 can decrease the expression of chemokines and constrain an inflammatory storm [10]. Additionally, a study has shown that the use of CCR (C–C chemokine receptor) antagonists, macrophage inflammatory protein (MIP)-2 antagonists, and other chemokine antagonists can inhibit the recruitment of neutrophils and limit the development and progression of myocarditis [16]. These discoveries suggest that early blockade of neutrophil recruitment can mitigate neutrophil infiltration, reduce the self-recruitment and activation of neutrophils, and tilt the immune response toward decreased inflammation.

### 2.2 Secretion of Cytokines Regulates the Defense Response in Myocarditis

Following the accumulation of neutrophils at the site of myocarditis injury, neutrophil TLRs recognize myocardial infection, degrade NF-κB inhibitor α (IκB-α), and activate the secretory vesicle pathway, Golgi–endoplasmic reticulum pathway, and cell lysis pathway implicated in cytokine release upon neutrophil activation [14]. Clusters of neutrophils recruited together generate cytokines, such as tumor necrosis factor-alpha (TNF-α), IL-1β, and interferon-γ (IFN-γ), whose functions encompass the regulation of the defense response and neutrophil degranulation [32]. The abundant infiltration of neutrophils in the heart and the extensive release of cytokines are the primary steps in myocarditis [33]. A disintegrin and metalloproteinase 17 (ADAM17) cleaves transmembrane TNF-α precursor (pro-TNF-α) into soluble TNF-α, thereby activating the inflammatory response in neutrophils and facilitating the release of TNF-α [34]. Subsequently, TNF-α released by neutrophils leads to a considerable increase in extracellular DNA release, forming DNA traps with monocytes and contributing to the elimination of myocarditis virus and/or myocardial tissue damage [14]. The elevated level of TNF-α is also associated with the progression of chronic myocarditis [35] and is supported by the demonstration of upregulated TNF-α level in the cardiac tissue of patients with chronic Chagas myocarditis, thereby facilitating the migration and infiltration of T lymphocytes into myocardial tissue and exacerbating inflammatory injury [36]. TNF-α can also intensify myocardial inflammatory responses by activating the P38 mitogen-activated protein kinase (MAPK) signaling pathway and upregulating the expression of other proinflammatory factors, such as IL-1β [37, 38]. Indeed, IL-1β is highly expressed in myocarditis and cooperates with TNF-α to promote neutrophil infiltration and NADPH oxidase activation, further amplifying inflammatory signals and inducing cardiomyocyte apoptosis [39]. An elevation in IL-1β induces the expression of lipocalin 2/neutrophil gelatinase B-associated lipocalin (Lcn2/NGAL), which is a strong myocarditis-inducing protein that is expressed in activated cardiac neutrophils [40]. Furthermore, IL-12 is critical in inducing and/or maintaining neutrophil functions following myocarditis [35]. IL-12 typically promotes the differentiation of Th1 cells and enhances the secretion of IFN-γ. Meanwhile, an imbalance in the Th1/Th2 ratio and elevated levels of IFN-γ has been confirmed to be closely associated with myocardial injury in viral myocarditis [4]. In myocarditis, IFN-γ can trigger immune responses from CD4^+^ T cells. As a pleiotropic cytokine, IFN-γ can enhance the killing of pathogens by immune cells and cooperate with other immune cells to eliminate virus-infected cells [41]. The immunomodulatory activity of IFN-γ also plays an intricate and vital role in determining the long-term antiviral status of the body. Meanwhile, IFN-γ overexpression in chronic myocarditis is closely related to the persistence of inflammation and the exacerbation of myocardial injury [4]. In addition, some cytokines, such as IL-17 and lymphocyte function-associated antigen-1 (LFA-1), enhance neutrophil infiltration and recruitment to heart damage sites by regulating the levels of neutrophil chemokines [42, 43].

In myocarditis, cytokines not only function independently but also drive the inflammatory process by forming complex positive feedback loops and cross-regulatory networks. TNF-α stimulates the release of IL-1β through the activation of p38 MAPK [44]. IL-1β and TNF-α collaboratively enhance ROS signaling, thereby accelerating ventricular remodeling. This multi-factor synergistic effect continuously amplifies the inflammatory response, serving as a central mechanism for the progression from myocarditis to dilated cardiomyopathy. Apart from cytokines, neutrophils can also secrete other molecules, such as Lcn2, which increase inflammatory cytokine levels and induce iron accumulation within cardiomyocytes, resulting in cardiomyocyte ferroptosis (Fig. 2) [43]. Overall, neutrophils possess diverse capabilities and are capable of releasing multiple cytokines that facilitate the development of myocarditis.

### 2.3 Secretion of Neutrophil Extracellular Traps is a Driver of Myocarditis

NETs are network structures containing DNA, histone, MPO, NE, and cathepsin G. Under myocarditis conditions, neutrophils recruited to the site of inflammation trigger the release of NETs via reticular tissue proliferation in a novel form of programmed cell death termed NETosis [12, 45]. The released DNA complexes with histones and granular proteins, forming a mesh-like structure in which histones play a crucial role in stabilizing the DNA and provide a scaffold for the assembly of antimicrobial proteins [46]. Numerous findings suggest that NETs are key contributors to the inflammatory cascade. Thus, exploring the role of NETs in myocarditis in depth may help understand the intricacies of the immune response. ADAMTS-1 disrupts extracellular matrix (ECM) homeostasis by degrading von Willebrand Factor (vWF) and aggrecan. The resulting ECM fragments can activate neutrophils and trigger the release of NETs. Midkine (MK), a key factor released by inflammatory cells to activate neutrophils, plays a crucial regulatory role in the formation of NETs, whereby MK binds to specific receptors on the surface of polymorphonuclear neutrophils (PMNs), and activates intracellular signaling pathways that direct PMNs to gather at the site of inflammation [47]. Low-density lipoprotein receptor–related protein 1 (LRP1) is a member of the low-density lipoprotein (LDL) receptor family that interacts with adhesion molecules in the β2 integrin family. LRP1 is expressed on the surface of PMNs and is capable of interacting with a variety of signaling molecules. MK mediates recruitment and adhesion of PMNs and promotes NET formation by binding to LRP1 [48]. Consequently, blocking MK might influence the activation of NETs and thereby impede the advancement of myocarditis, offering novel and promising treatment alternatives for patients with myocarditis.

PAD4 is a calcium-dependent enzyme that recognizes and binds arginine residues. In neutrophils, PAD4 is mainly located in the cytoplasm, nucleus, and azurophilic granules. This distribution allows PAD4 to function in different cellular regions. PAD4 in the cell nucleus promotes chromatin depolymerization by modified histones, releasing a DNA network structure that captures pathogens. PAD4 regulates signaling pathways in the cytoplasm by modifying non-histone proteins. PAD4 can also be secreted extracellularly through exosomes to mediate intercellular communication [49]. Thus, it has been proposed that NET release is decreased after the activation of neutrophils in PAD4 knockout mice in viral myocarditis (VM). This finding suggests that NETs can cause heart necrosis and inflammation in myocarditis, and the activation of PAD4 in the cell nucleus is a marker for the onset of NETosis in myocarditis [50]. GSK484, a PAD4 inhibitor, can ameliorate the inflammatory response of myocarditis by inhibiting PAD4 activation and terminating NETosis in the initial stage of myocarditis (Fig. 2) [51]. This discovery could constitute a potential therapeutic strategy for treating myocarditis.

However, the development of therapeutic drugs for myocarditis caused by NETs encounters numerous challenges. Regarding drug delivery, it is essential to enhance cardiac targeting to penetrate the myocardial tissue barrier effectively while minimizing systemic exposure. Concerning the risk of off-target effects, MK blockers might interfere with physiological processes such as tissue repair and angiogenesis, whereas PAD4 inhibitors could induce epigenetic disorders due to their involvement in histone modification. Toxicity reduction can be achieved through structural optimization or localized administration. Thus, the timing of treatment necessitates a dynamic balance. Indeed, early inhibition of NETs may compromise antiviral immunity, while late intervention has limited efficacy in reversing fibrosis [2]. Therefore, it is crucial to define the treatment window accurately in conjunction with biomarkers. Despite preclinical studies demonstrating the anti-inflammatory potential of PAD4 inhibitors and MK blockers, the clinical translation of these compounds remains hindered by obstacles, such as the high heterogeneity of myocarditis and recruitment difficulties for trials of rare diseases. In the future, multi-omics technology should be employed to analyze the spatiotemporal dynamics of NETs, and combination strategies with existing therapies should be explored to enhance both efficacy and safety. During NETosis, citrullinated histones H3 and H4 and neutrophilic nucleosomes are released into the extracellular space along with serine proteases and other granulocytes [50], and neutrophils are accompanied by an increased presence of MPO and NE in cells as well as in interstitial staining debris, which can serve as evidence of NET formation during the process of secreting various proteases and degranulation through NETosis [52]. Therefore, in the pathogenesis of myocarditis, the content of MPO and NE can indirectly reflect the progression of myocarditis and provide auxiliary means for the examination of myocarditis.

A previous study has shown that NETs exhibit anti-inflammatory effects, including the trapping of pathogens and the limitation of viral spread [17]. However, recent studies have shown that while NETs are critical in eradicating infection, an excess of NETs can transfer autoantigens, including DNA and histones, to the host immune system and represent prime therapeutic targets for myocarditis. Weckbach *et al*. [53] confirmed that the inhibition of NETs, which can be detected in myocardial tissues of patients and mice with autoimmune myocarditis, reduced inflammation in the acute phase of the disease. In addition, Carai *et al*. [50] further verified that inhibiting NETosis in mice at the acute stage of viral myocarditis could effectively reduce cardiac inflammation and improve the pathological phenotype. Thus, NETs are largely involved in the pathogenesis of myocarditis and drive the development of cardiac inflammation. NETosis shows characteristic changes in different types of myocarditis. In viral myocarditis, viral RNA and DNA enhance neutrophil NETosis by activating the TLR–p38 MAPK pathway. The released NETs directly damage cardiomyocytes and promote the spread of the virus [3]. In autoimmune myocarditis, NETs activate B cells and Th17 cells by exposing citrullinated histones and MPO as autoantigens, driving antibody-dependent and T cell-mediated myocardial injury. In drug-induced myocarditis, the TNF-α/IFN-γ storm activates the Nox2–ROS pathway, resulting in a sharp increase in NETosis and triggering microvascular embolism. The released histones and S100A8/A9 induce M1 polarization in macrophages (proinflammatory phenotype), inhibit Treg function, and activate cardiac fibroblasts to promote fibrosis [54, 55]. Thus, NETosis forms a vicious cycle with immune cells.

In the pathology of myocarditis, excess NETs and their components augment the proinflammatory function of neutrophils, thereby enhancing the secretion of chemokines and cytokines. NETs participate in autoimmunity as inflammatory inducers, recruiting neutrophils and other infiltrating immune cells to the heart and exposing cardiac tissue to an immune attack. Senescent neutrophils exhibit elevated mitochondrial ROS levels and enhanced leakage of mitochondrial DNA in viral myocarditis. These components are capable of augmenting the antigen-presenting function of dendritic cells by activating pattern recognition receptors, such as TLR-9, and facilitating the cytotoxic responses of CD8⁺ T cells [56]. In autoimmune myocarditis, the autoantigens carried by NETs can disrupt immune tolerance and promote the pathological damage mediated by Th17 cells [57, 58]. Moreover, NETs can also activate mononuclear and macrophage differentiation and phagocytosis through the inflammasome pathway, thereby enhancing the inflammatory response and exacerbating heart injury [50]. Research indicates that IL-37 can inhibit the phosphorylation of NF-κB/IκBα, thereby limiting the activation of the inflammasome pathway in myocarditis mice and preventing the formation of NETs in myocardial tissue [59]. In addition, DNase I degrades NETs–DNA, reducing inflammatory factors and significantly inhibiting cancer-related myocarditis and myocardial stress [60]. NETs are not only the body’s defense line against infection, but also important regulators of the inflammatory response. Exploring NETosis and its underlying mechanisms, which cause autoimmune diseases, may identify new therapeutic targets and have crucial clinical implications.

## 3. Neutrophil Phagocytosis

Phagocytosis is a crucial step for neutrophils to engulf and eliminate pathogens after patients develop myocarditis. Pathogens are eliminated through both oxygen-dependent (generation of reactive oxygen species) and oxygen-independent (intracellular degranulation and release of enzymatic substances) mechanisms within neutrophils [61]. In myocarditis, increased degranulation results in tissue damage and excessive activation of the inflammatory response [14]. Excess ROS can cause oxidative stress and damage cellular structures, thus aggravating myocardial damage. The release of NE and MPO can degrade the myocardial extracellular matrix, destroy myocardial structure, and promote fibrosis and scar formation. ADAM8 regulates the phagocytic activity of neutrophils and their capacity to clear pathogens by cleaving the extracellular domain of integrins. Abnormal ADAM8 activity may impair phagocytic function, thereby exacerbating viral spread [62]. Research on the phagocytosis of neutrophils may provide a basis for individualized treatment of myocarditis.

### 3.1 Neutrophil Degranulation Damages Cardiomyocytes

**Fig. 3.**
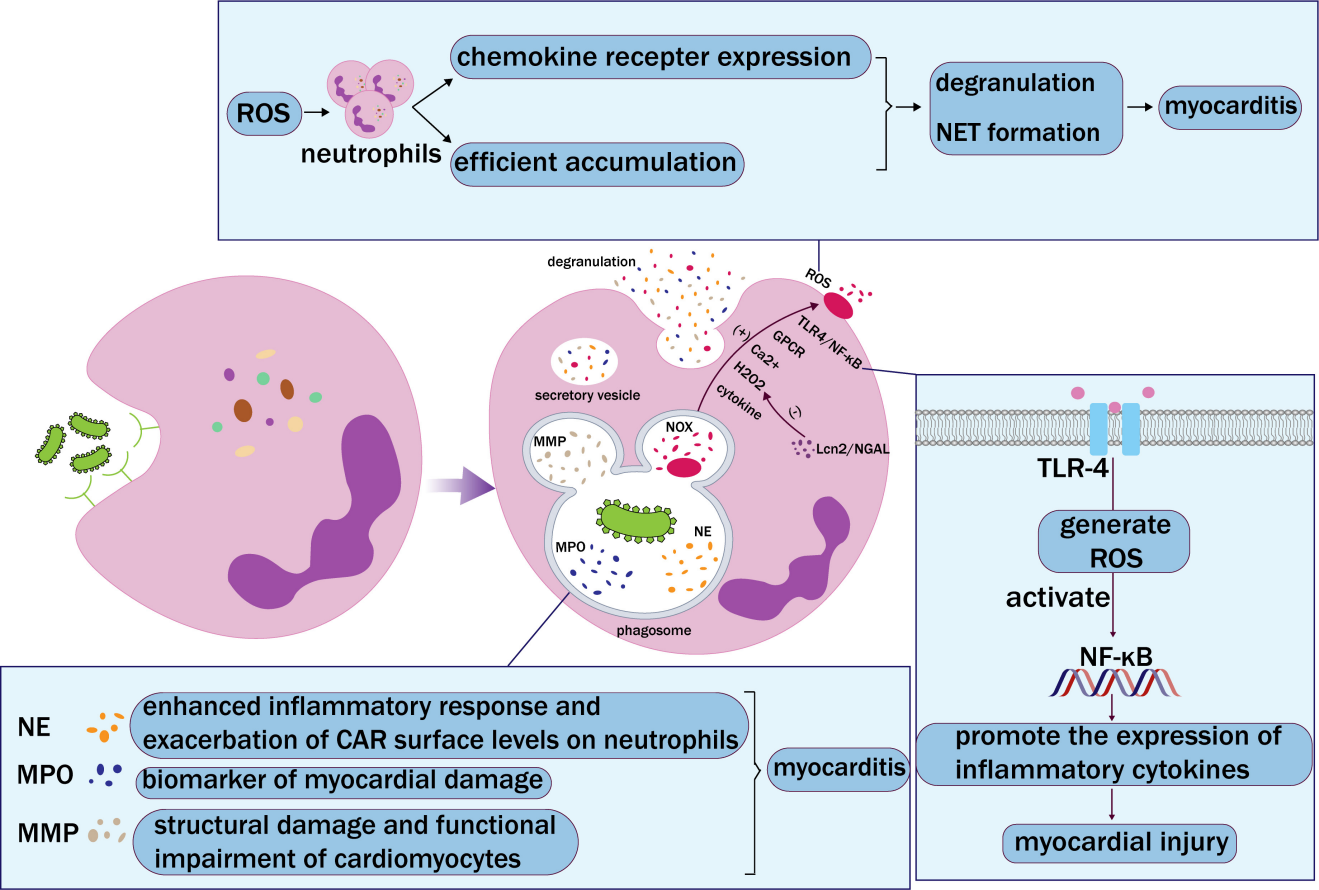
**Neutrophil phagocytosis**. Neutrophils recognize and bind pathogens through receptors on their surface, which are then engulfed by phagosomes. Phagosomes and lysosomes fuse and degranulate to release neutrophil elastase (NE), myeloperoxidase (MPO), matrix metalloproteinase (MMP), and NADPH oxidase (NOX), and finally form extracellular vesicles. NE, MPO, and MMP accelerate myocarditis in different ways. Activation of NOX mediates the production of reactive oxygen species (ROS). In addition, chemokines, Toll-like receptor 4 (TLR-4)/nuclear factor kappa-light-chain-enhancer of activated B (NF-κB) pathway activation, G protein-coupled receptors (GPCRs), Ca^2+^, and hydrogen peroxide (H_2_O_2_) can induce the production of ROS. Lipocalin 2/neutrophil gelatinase B-associated lipocalin (Lcn2/NGAL) can reduce ROS production by inhibiting H_2_O_2_ activity. ROS enhances the function of chemokine receptors on the surface of neutrophils and enables neutrophils to accumulate more efficiently at the site of myocardial injury. In this way, degranulation and neutrophil extracellular trap (NET) formation can be increased. ROS induce the occurrence and development of myocarditis.

The degranulation of neutrophils is a key mechanism underlying their bactericidal and anti-inflammatory responses. In the context of myocarditis, neutrophils release various enzymes and antimicrobial substances through degranulation, which can directly kill invading pathogens while also potentially causing damage to myocardial cells. Neutrophils form four consecutive types of granules during maturation (Fig. 3). Primary granules serve as storage sites for elastase, MPO, cathepsin, and defense proteins. Secondary granules mainly encompass NADPH oxidase, lactoferrin, and matrix metalloproteinase-9 (gelatinase). Tertiary granules are abundant in gelatinase, a kind of MMP, but lack lactoferrin. Secretory vesicles are membrane-wrapped vesicles rich in proteins and peptides [61]. Among them, NE, a serine protease produced by neutrophils, plays a crucial role in the pathogenesis of myocarditis. NE can activate proinflammatory cytokines, enhance the adhesion and migration of neutrophils, and increase both the number of heart-infiltrating inflammatory cells and the expression of genes related to inflammation. Akt signaling has also been shown to attenuate the apoptosis of cardiomyocytes. NE can enhance myocardial injury by inhibiting Akt signal transduction [63].

The coxsackie and adenovirus receptor (CAR) is the key receptor for eponymous viruses capable of causing myocarditis. The CAR is a target for neutrophil elastase-mediated shedding, as demonstrated by *in vitro* assays [64]. NE can aggravate myocarditis viral infection by exacerbating CAR surface levels on neutrophils [64]. Based on the activity of NE, responsive drug delivery systems can be designed to break through the non-specific distribution bottleneck of traditional anti-inflammatory drugs and provide a new therapeutic paradigm for myocarditis. MPO is a biomarker of myocardial damage, and its activity and expression levels are heightened in patients with myocarditis. MPO can also catalyze the formation of hypochlorous acid, which can damage myocardial cell membranes [65]. Therefore, an elevated MPO level in blood tests may indicate inflammatory activity and contribute to the diagnosis of myocarditis [15]. MMPs play a double-edged role in the pathological process of myocarditis. On the one hand, MMPs help clear infections; on the other hand, the excessive release of MMPs and other inflammatory mediators can lead to structural damage and functional impairment of cardiomyocytes, thus exacerbating the condition of myocarditis [66]. In addition, ROS, NETs, and other inflammatory mediators produced by degranulated neutrophils can also lead to damage, apoptosis, and necrosis of myocardial cells [53].

Neutrophil degranulation alters the composition and properties of the extracellular matrix, which in turn affects the activation, recruitment, and further degranulation of neutrophils through multiple mechanisms, forming a complex positive feedback regulatory network. Degradation of the extracellular matrix releases endogenous chemokines, such as IL-8 and CCL2, which can further attract more neutrophils to infiltrate the inflammatory site [67]. In addition, new binding sites will also be exposed. These sites can bind to integrins and other adhesion molecules on the surface of neutrophils, enhancing the adhesion and migration capabilities of neutrophils [68]. Alterations in the extracellular matrix can activate local inflammatory signaling pathways, such as the NF-κB and MAPK pathways, which can further promote the activation and degranulation of neutrophils [69]. Therefore, regulating the degranulation of neutrophils and reducing their damage to cardiomyocytes may become a crucial strategy for treating myocarditis.

### 3.2 Increased ROS Production Damages Cellular Structures in Myocarditis

In myocarditis, neutrophils are significant sources of oxidative stress and can produce a range of oxidant molecules upon activation [70]. Cytokines, agonists of Toll-like receptors or G protein-coupled receptors (GPCRs), and intracellular Ca^2+^ increases are known to activate ROS production through NADPH oxidase (NOX) [71]. Additionally, the TLR4/ROS/NF-κB pathway is a crucial pathway for ROS production in myocarditis. Evidence suggests that TLR4/NF-κB is involved in myocardial injury during chronic stress [72]. NF-κB, induced and activated by TLR4, is involved in gene expression in myocarditis and produces ROS through multiple mechanisms to promote oxidative stress [73]. In the early stage of myocarditis, ROS participates in signal transduction and cellular defense mechanisms [74]. ROS can enhance the function and expression of chemokine receptors on the surface of neutrophils, enabling neutrophils to accumulate more rapidly and efficiently at the site of myocardial injury. In addition, ROS plays a significant role in the degranulation of neutrophils and the release of various bactericidal substances and inflammatory mediators [39]. This approach effectively eradicates pathogens, thereby mitigating myocardial infection and offering protective effects to the myocardium. ROS also prevents the spread of myocarditis by facilitating the formation of NETs [75], which provides a favorable environment for myocardial tissue repair. However, excess ROS can cause increase of oxidative stress and damage cellular structures, exacerbating tissue damage in myocarditis [14]. ROS can also promote the degranulation of neutrophils and the release of inflammatory factors, thereby exacerbating the inflammatory response. The excessive production of ROS can lead to the disruption of intracellular signaling pathways, further activating the NF-κB signaling pathway, inducing the release of inflammatory factors, forming a vicious cycle, and enhancing the inflammatory response in myocarditis (Fig. 3). Intervening in the ROS produced by neutrophils may provide new insights for treating myocarditis. A recent study revealed that Lcn2/NGAL offers protection against the toxicity of hydrogen peroxide (H_2_O_2_), an inducer of oxidative stress resulting from the generation of reactive oxygen species, thereby providing a potentially beneficial impact in ameliorating the toxicity induced by oxidative stress [43].

## 4. The Functional Synergy Network of Neutrophils

Neutrophils are a major component of the immune system and perform an important defensive role. In the pathological process of myocarditis, neutrophil secretion and phagocytosis constitute a complex immune response network. The functional synergy network of neutrophils not only involves the actions of neutrophils themselves but is also closely related to the interactions with other immune cells.

### 4.1 Relationship Between Secretion and Phagocytosis in Myocarditis

**Fig. 4.**
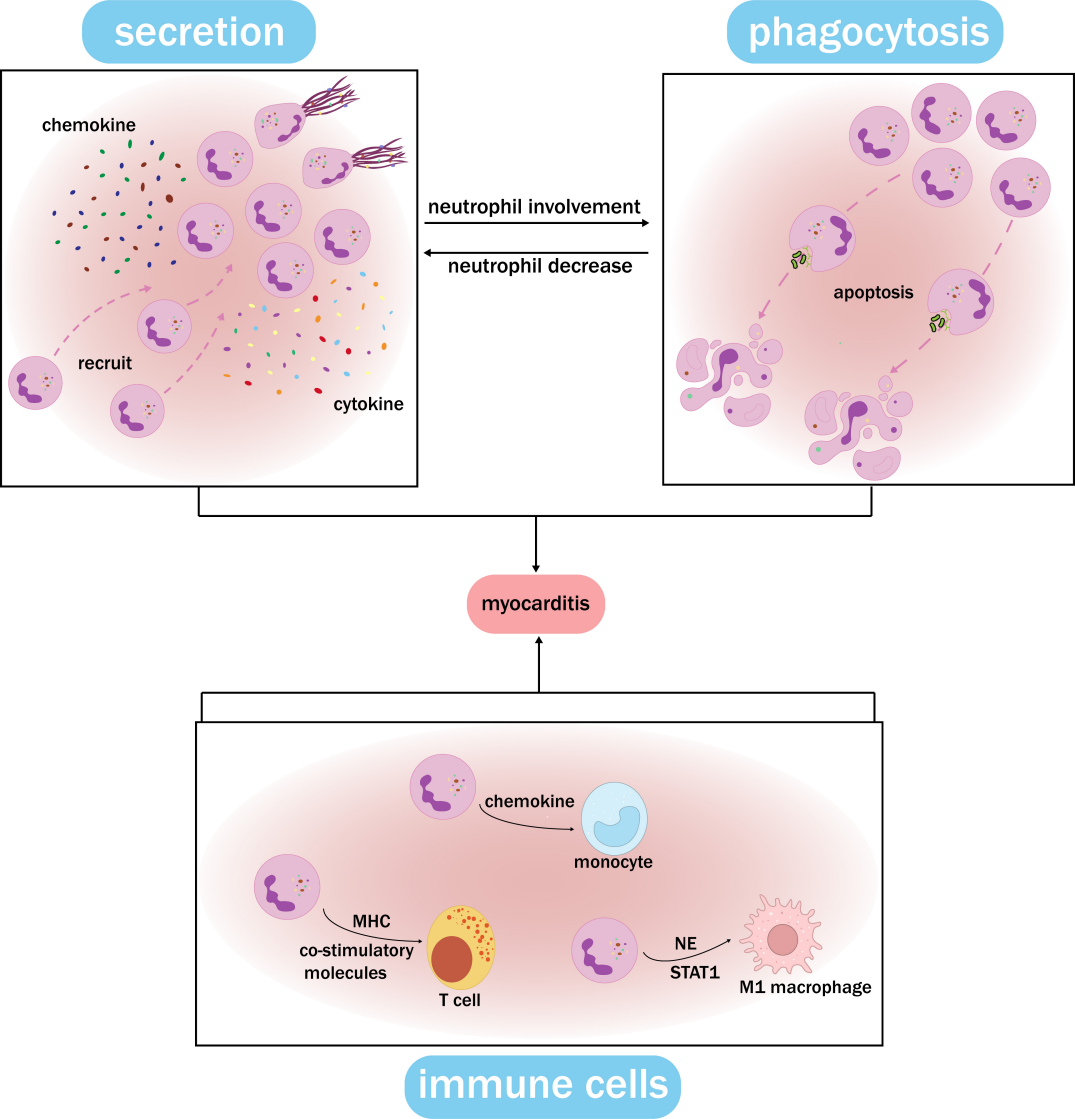
**Functional synergy network of neutrophils**. Neutrophils at the site of inflammation secrete chemokines and cytokines, inducing the formation of NETs and the recruitment of cells. After accumulating, neutrophils undergo phagocytosis, which leads to apoptosis. The remaining neutrophils at the inflammation site show increased secretion and self-recruitment, creating a feedback loop. Meanwhile, neutrophils recruit and activate monocytes, T cells, macrophages, and other immune cells through distinct pathways, and this interaction plays a crucial role in myocarditis. The functional synergy network of neutrophils can contribute to myocarditis by ensuring the stability of the neutrophil count, exerting their proinflammatory role, and damaging cardiomyocytes. MHC, major histocompatibility complex; NE, neutrophil elastase; NET, neutrophil extracellular trap.

In myocarditis, secretion and phagocytosis by neutrophils are not two isolated functions, but rather a unified entity. Neutrophils at the inflammatory site facilitate the recruitment and activation of immune cells by secreting chemokines and cytokines [14]. Once neutrophils accumulate, these cells commence phagocytosis, engulfing and eliminating pathogens [33]. After phagocytosis, neutrophils typically undergo programmed cell death (apoptosis) and are cleared by other immune cells [76]. The remaining neutrophils at the inflammatory site exhibit more intense secretion and self-recruitment [56]. In myocarditis, the secretion and phagocytosis of neutrophils constitute an effective cycle (Fig. 4). This not only ensures the stability of the neutrophil count at the inflammatory site but also maintains the persistence of neutrophil activity at all times. However, if the inflammation is not controlled in a timely manner, this relationship may damage the heart. Excessive phagocytosis and secretion induce the release of a large number of inflammatory mediators, which damage cardiomyocytes and attract additional inflammatory cells, forming an “amplification loop” of inflammation. This allows neutrophils to continuously exert their proinflammatory role, thereby enhancing the occurrence and development of myocarditis.

### 4.2 Interaction Between Neutrophils and Other Immune Cells

Neutrophils not only play an independent role in the pathological progression of myocarditis but also interact closely with other immune cells. Research has found that in the early stage of myocarditis, neutrophils can promote the migration and activation of monocytes by secreting chemokines, thereby enhancing the local immune response [33]. In addition, neutrophils can directly activate T cells by expressing major histocompatibility complex (MHC) molecules and co-stimulatory molecules, promoting the occurrence of specific immune responses. This interaction may play a significant role in the chronic stage of myocarditis, leading to persistent immune activation and myocardial damage [77]. Neutrophils can also induce polarization of M1 macrophages by releasing NE, thereby enhancing their inflammatory response, which can be achieved in macrophages through the STAT1 signaling pathway. This interaction may play a key role in the pathogenesis of myocarditis, leading to further damage to cardiomyocytes. Neutrophil secretion and phagocytosis, as well as interaction with other immune cells, are an indispensable part of the pathological process of myocarditis, and have important clinical value for treating myocarditis [78].

## 5. Conclusion and Future Perspectives

Targeting secretion and phagocytosis by neutrophils shows great promise for the treatment of myocarditis. Neutrophils, due to their roles in cytokine secretion and innate immunity against viruses, are an ideal target for therapy. However, at present, neutrophil-related drugs are mostly at the clinical trial stage and lack corresponding diagnostic and treatment guidelines, which limits their scope of application. Indeed, in the future, by conducting more in-depth research into the mechanisms of neutrophil phagocytosis and secretion, a better understanding of the pathogenesis of myocarditis caused by neutrophil-released substances will result, which will lead to highly effective solutions to the current challenges and treatment gaps in myocarditis.
